# Femoral Nerve Palsy Post Total Hip Arthroplasty (THA) via a Posterolateral Approach

**DOI:** 10.7759/cureus.50771

**Published:** 2023-12-19

**Authors:** Ali A AlGhufaily, Abdullah I Alshunaifi, Jwaher S AlHarbi

**Affiliations:** 1 Department of Orthopaedics, Security Forces Hospital, Riyadh, SAU; 2 Department of Orthopaedics, Princess Nourah Bint Abdulrahman University, Riyadh, SAU

**Keywords:** hip surgery, developmental dysplasia of the hip ( ddh ), total hip arthroplasty, femoral nerve palsy, posterolateral approach

## Abstract

Femoral nerve palsy (FNP) is a debilitating and relatively rare complication of total hip replacement, which can worsen the functional prognosis. Various surgical approaches, including posterior, lateral, and anterior, are employed in total hip arthroplasty (THA), and the chosen approach can influence which nerve is affected. There is currently a lack of recent research on the prevalence of FNP and its typical course of recovery. In this clinical case, we report a rare incidence of FNP that presented as a complication of primary THA in a patient with end-stage osteoarthritis secondary to developmental dysplasia of the hip (DDH). A 35-year-old female presented with groin pain and restricted movement in her hip joint. She had a history of undergoing complex open-reduction surgery for hip dysplasia. During the physical examination, a positive Trendelenburg gait was identified, along with end-stage osteoarthritis (OA) secondary to the dysplasia. She subsequently underwent THA using a posterolateral approach. Following the procedure, she experienced neurological symptoms, leading to the diagnosis of FNP, a rare occurrence.

## Introduction

Femoral nerve palsy (FNP) is a rare complication of total hip arthroplasty (THA), with a documented incidence ranging between 0.08% and 7.6% [1]. Possible causes include compression due to retractor placement or the development of hematomas, as well as traction, laceration, ischemia, or thermal damage [2]. It typically presents as excruciating pain in the inguinal area, accompanied by iliac fossa tenderness. However, the most noticeable sign is weakness of the quadriceps muscles, which may make walking difficult [3]. We report the case of a 35-year-old female who complained of groin pain and limited hip joint movement. She had a history of complicated open-reduction surgery for developmental dysplasia of the hip (DDH). A physical examination revealed a positive Trendelenburg gait and end-stage osteoarthritis (OA) secondary to dysplasia. She underwent THA via the posterolateral approach. Post-surgery, she experienced neurological symptoms and was diagnosed with FNP, a rare occurrence for the posterolateral approach.

## Case presentation

**Figure 1:**
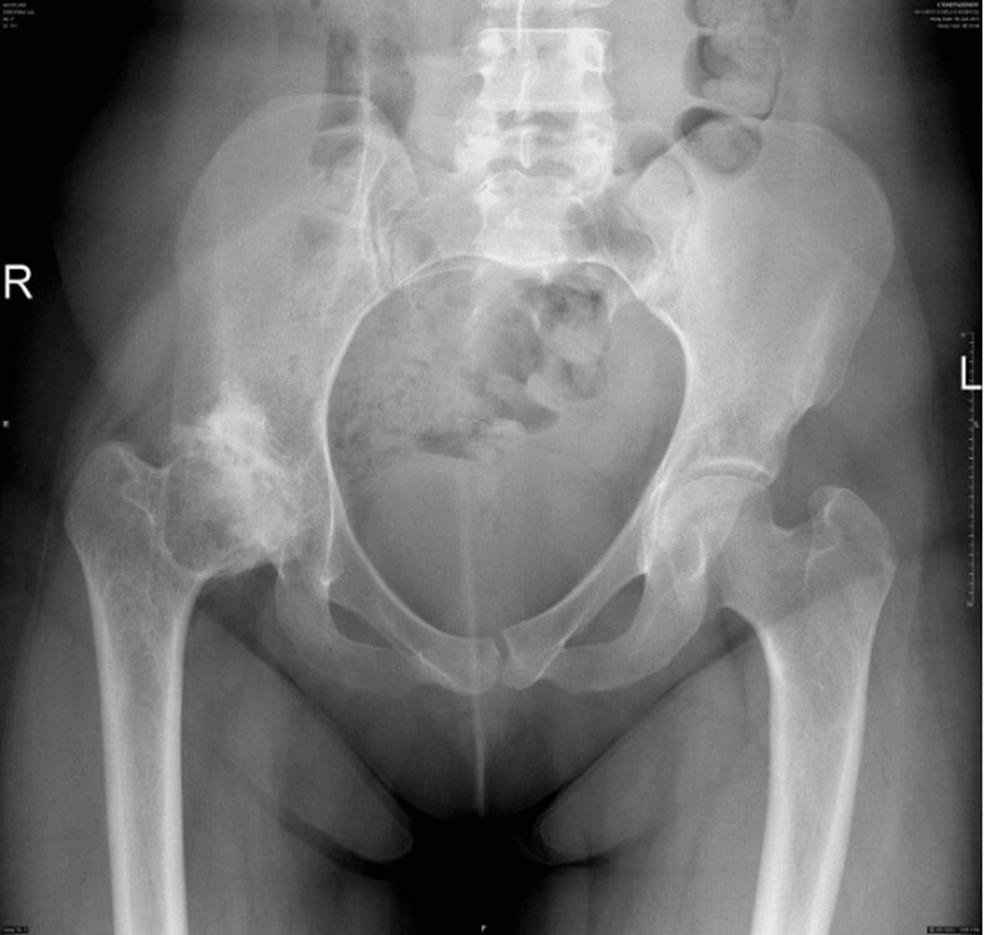
AP X-ray showing hip at the time of presentation. AP: Anteroposterior.

**Figure 2:**
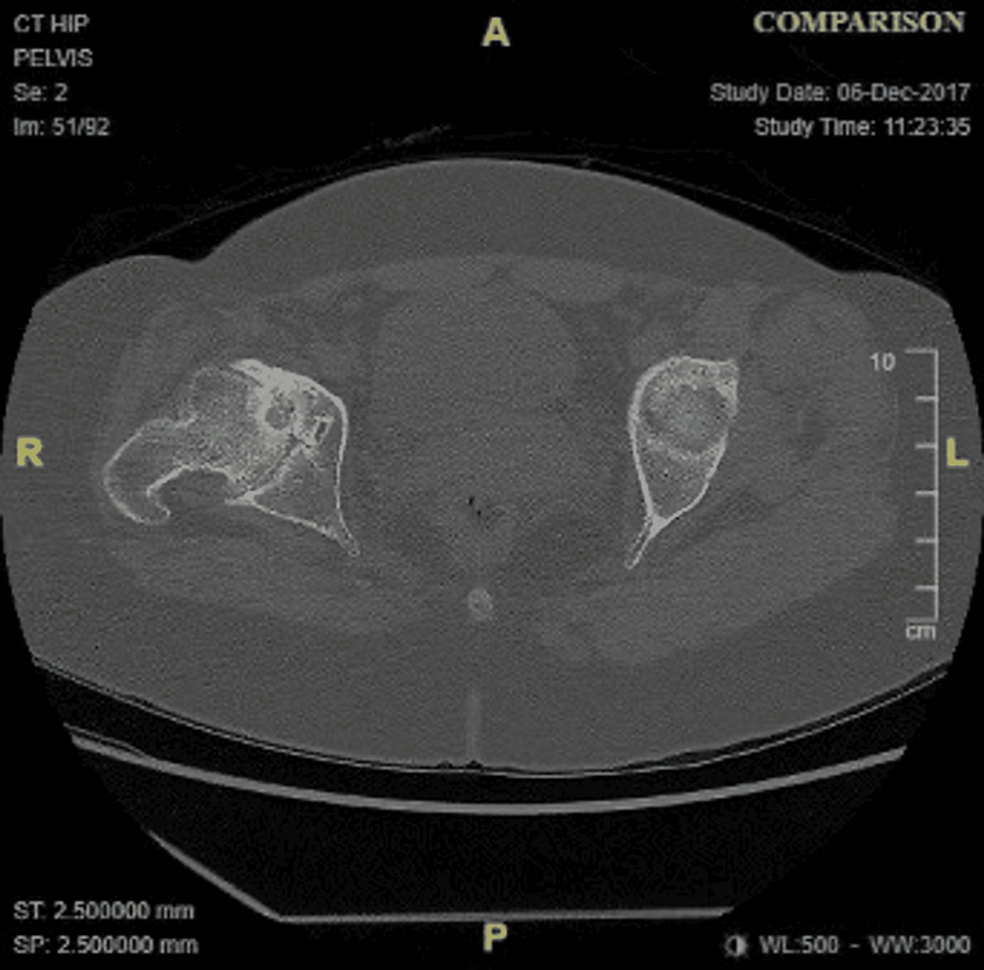
Axial CT showing the hip at the time of presentation.

A 35-year-old female patient with a restricted range of motion in her hip presented to our clinic with subtle groin pain that began three years prior to her visit. She had previously undergone open reduction surgery for DDH (Figures 1-2). The patient was then followed up at another hospital until she reached 11 years of age. However, no further surgical procedures were performed. The preoperative assessment revealed a 2.5 cm leg length discrepancy and a positive Trendelenburg gait but no signs of neurovascular injury. Plain radiographic imaging showed end-stage OA secondary to dysplasia. The patient was scheduled for THA. A cementless total hip replacement was performed via the posterolateral approach. The patient was positioned in left lateral decubitus. The anterior pelvis peg was positioned at the level of the pubic symphysis, and the posterior pelvis peg was positioned above the gluteal cleft. The Zimmer Continuum acetabular hemispherical cup system, combined with the cementless Zimmer Avenir femoral stem and screw fixation, was used in the posterior superior and posterior inferior quadrants of the acetabulum. Bone grafts were applied for superior coverage of the acetabulum and medial augmentation. Three retractors were used during the procedure to expose the acetabulum: a posterior acetabular retractor over the posterior rim, an anterior acetabular retractor on the anterior rim, and a Steinman pin inserted into the roof of the acetabulum to retract the abductors.

**Figure 3:**
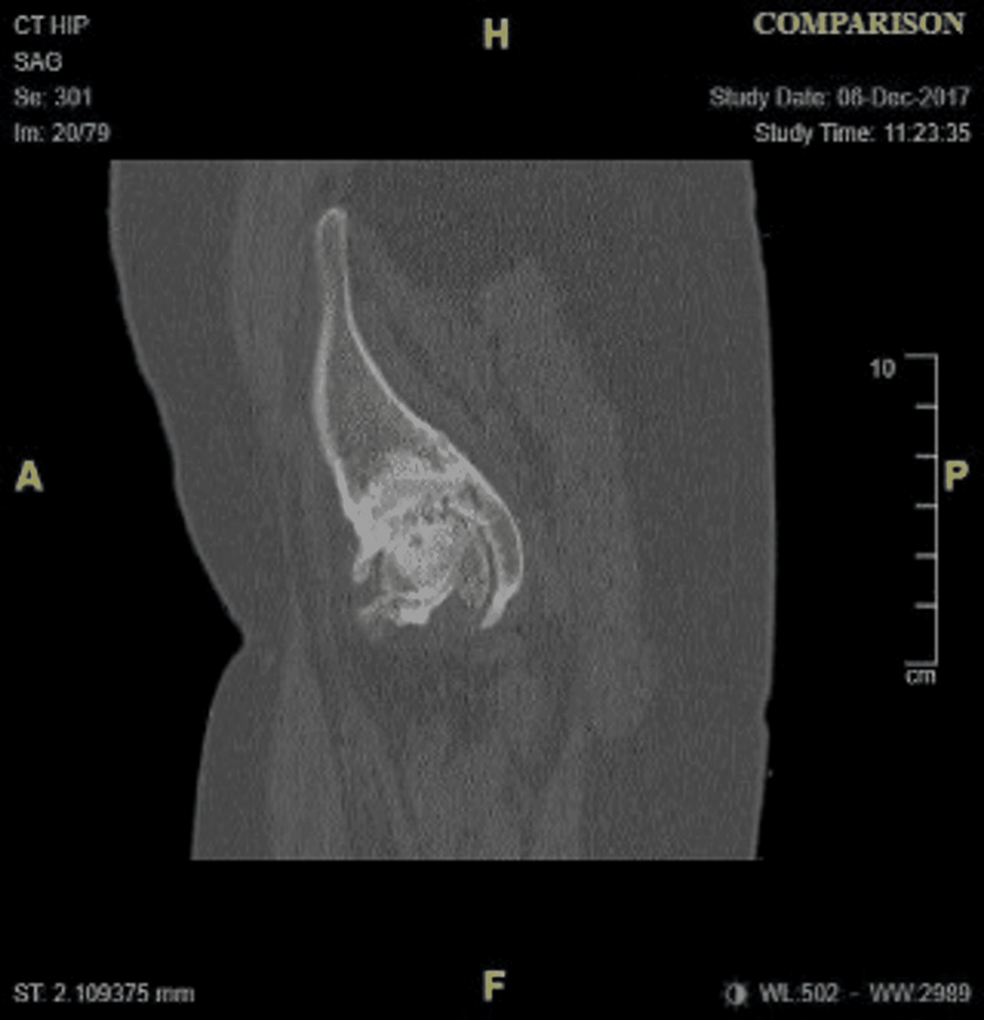
Sagittal CT showing the hip at the time of initial presentation.

On postoperative day 1 (Figure 3), the patient complained of anterior and medial thigh paresthesia and weakness in the quadriceps muscle. There were no signs of hematoma collection. It was decided to manage the patient conservatively and closely monitor her in the clinic, with plans to proceed with a nerve conduction study if there were no signs of recovery after three months. The patient returned to the clinic four weeks after the operation with the same complaints of anterior and medial thigh paresthesia and difficulty climbing stairs. Physical examination revealed a quadriceps-deficient gait.

**Figure 4:**
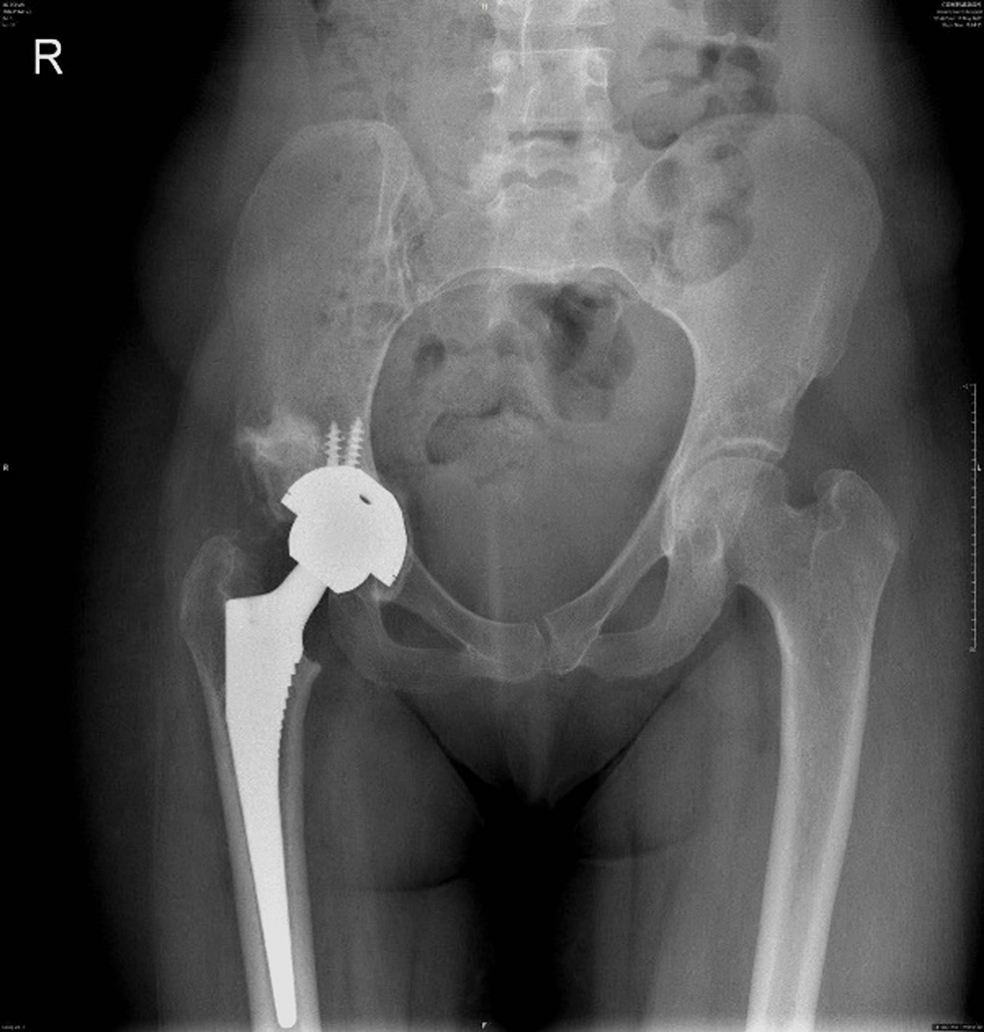
AP X-ray of the hip post operatively. AP: Anteroposterior.

Her assessment of muscle power revealed 5/5 for the iliopsoas muscle, 3/5 for the hip adductors, and 1/5 for the quadriceps muscle power. She was unable to perform a straight leg raise. There was decreased sensation over the inner, medial, and anteromedial thighs. The patient was closely followed in the clinic for femoral and obturator nerve palsy. Three months after the surgery, the patient was able to walk with assistance, but she was still unable to perform the straight leg raise test. Five months post-surgery, all motor and sensory side effects had completely resolved (Figure 4).

## Discussion

Dislocation, looseness, infection, embolisms, blood clotting, vascular injury, fractures, and nerve injury (nerve palsy) are some complications associated with THA. Infection and dislocation may lead to revision THA in the short term, while the rate of revision THA due to loosening increases over long-term follow-up [4]. Furthermore, it has been reported that cement entrapment can result in long-lasting femoral nerve impairment [5].

Nerve injury following THA is an uncommon complication. The incidence is lower in primary THA compared to revision cases [1]. This complication can result in pain and weakness, adversely affecting patient satisfaction and outcomes due to its debilitating effects. Paralysis of the obturator nerve is rare; the sciatic nerve, implicated in over 80% of cases, is the most commonly affected, followed by the femoral nerve and the combination of the sciatic and femoral nerves [6-7]. Given that femoral nerve palsy generally has a better prognosis than sciatic nerve palsy, the affected nerve seems to be significant [8].

According to reports, the likelihood of developing nerve palsy after THA varies depending on the surgical approach [9]. These approaches include the anterior approach [10], lateral approach [11] (consisting of anterolateral (Watson-Jones) and direct lateral (Hardinge)), and posterior approach [12] (encompassing posterolateral and posterior) [13]. However, the best strategy remains a subject of ongoing debate. The posterior approach has long been linked to nerve damage. Weale AE et al. [14] found no evidence to support earlier reports linking nerve injury with the posterior approach when comparing the frequency of nerve injury after initial total hip replacement utilizing either a posterior or a direct lateral approach. Interestingly, their study found the lateral approach to be associated with nerve palsy [14]. Additionally, Fleischman AN et al. reported that anterior hip surgery carries a 14.8-fold increased risk of fracture compared to other approaches [2].

In this clinical case, we describe an uncommon occurrence of femoral nerve palsy as a consequence of initial THA executed using the posterolateral approach. Numerous studies indicate that female patients with DDH are more susceptible to nerve damage following total hip replacement, as was the case in our report [5,14,15]. The etiology of this complication is often unclear, with potential causes including compression from retractor placement, development of hematomas, traction, laceration, ischemia, or thermal damage [2]. Iliacus hematomas, especially in patients on anticoagulants, are another known mechanism of femoral nerve injury [16]. To exclude the possibility of an expanding lesion or swelling due to a hematoma, a CT scan of the pelvic region is recommended [3].

During total hip replacement, the use of acetabular retractors has been linked to injuries to the femoral and obturator nerves. It is hypothesized that the tip of the anterior retractor, passing superficially to the iliopsoas, can compress the femoral nerve. Similarly, when retracted, the tip of an inferior retractor may move laterally, potentially coming into contact with the obturator nerve as it pierces the obturator membrane medially to the nerve, potentially causing nerve palsy [17]. In our case, as described, the retractors were properly positioned. However, we recommend avoiding vigorous traction, which we believe played a significant role in the development of femoral and obturator nerve palsy in our patient.

Femoral neuropathy after THA usually manifests as excruciating pain in the inguinal area, along with iliac fossa tenderness. However, the most noticeable signs are a decreased patellar reflex and a weakness of the quadriceps muscles, which may make walking difficult [3]. For the prognosis and the likelihood of recovery, electroconductive studies are necessary to ascertain the extent of the lesion [18]. The likelihood of neurological recovery depends on the extent of nerve damage. Compared to sciatic nerve palsy, FNP recovers more predictably and with less disability [6]. According to the study by Fleischman AN et al. [2], most patients do not start to recover significantly from FNP until more than six months after surgery. It further concluded that in less than two years, most patients can be expected to recover nearly fully with only minor motor deficits [2]. A study by Simmons C Jr. et al. [19] reports 10 patients with THA-related FNPs who fully recovered without experiencing any long-term disability [19]. Currently, conservative management remains the gold standard of care for FNP due to the lack of a clear management protocol in the literature [20]. Based on the available literature, which concludes that most patients recover completely without further intervention, we decided to treat the patient conservatively.

## Conclusions

To conclude, FNP is a rare complication of THA that can be treated conservatively. We further conclude that proper placement of retractors during the procedure is important in determining the postoperative complications of THA. We do not recommend investigating FNP with a nerve conduction study until three months postoperatively in patients with no signs of recovery to assess the severity of the damage and prognosis.
